# Microbial Lipid Based Biorefinery Concepts: A Review of Status and Prospects

**DOI:** 10.3390/foods12102074

**Published:** 2023-05-22

**Authors:** Jonilson de Melo e Silva, Luiza Helena da Silva Martins, Débora Kono Taketa Moreira, Leonardo do Prado Silva, Paula de Paula Menezes Barbosa, Andrea Komesu, Nelson Rosa Ferreira, Johnatt Allan Rocha de Oliveira

**Affiliations:** 1 Program of Food Science and Technology, Federal University of Pará (UFPA), Belém 66075-110, PA, Brazil; 2 Faculty of Food Science and Technology, Rural Federal University of the Amazon, Belém 66077-530, PA, Brazil; 3 Federal Institute of Brasília—IFB/Campus Gama, Brasília 72429-005, DF, Brazil; 4 Department of Food Science and Nutrition, Faculty of Food Engineering (FEA), State University of Campinas (UNICAMP), Campinas 13083-862, SP, Brazil; 5 Department of Food, Technical College of Campinas, University of Campinas, Campinas 13083-862, SP, Brazil; 6 Department of Marine Sciences (DCMar), Federal University of São Paulo (UNIFESP), Santos 11070-100, SP, Brazil; 7 Faculty of Food Engineering, Technology Institute, Federal University of Pará (UFPA), Belém 66077-000, PA, Brazil; nelson.ufpa@gmail.com; 8 Nutrition College, University of Pará, Augusto Correa Avenue, 01, Belém 66075-110, PA, Brazil

**Keywords:** lignocellulosic, biomass, lipids, biorefinery, single-celled oils

## Abstract

The use of lignocellulosic biomass as a raw material for the production of lipids has gained increasing attention, especially in recent years when the use of food in the production of biofuels has become a current technology. Thus, the competition for raw materials for both uses has brought the need to create technological alternatives to reduce this competition that could generate a reduction in the volume of food offered and a consequent commercial increase in the value of food. Furthermore, the use of microbial oils has been studied in many industrial branches, from the generation of renewable energy to the obtainment of several value-added products in the pharmaceutical and food industries. Thus, this review provides an overview of the feasibility and challenges observed in the production of microbial lipids through the use of lignocellulosic biomass in a biorefinery. Topics covered include biorefining technology, the microbial oil market, oily microorganisms, mechanisms involved in lipid-producing microbial metabolism, strain development, processes, lignocellulosic lipids, technical drawbacks, and lipid recovery.

## 1. Introduction

### 1.1. Lignocellulosic SCO (Single-Cell Oil)

The biorefinery can be defined as a promising path for an industry based on the use of biomass as an input for the production of high value-added products, so biorefineries basically aim to transform biomass into biofuels and/or fine chemical products. Such technologies can be considered emerging. The bottleneck is to achieve a less costly and more sustainable way for such a transformation of biomass to occur, as the products produced can be applied in various industries, being highly versatile [1]. In addition, the biorefinery has a wide range of possibilities for applying and obtaining products, with a variety of products known as platform chemicals, such as glycerol, in addition to biofuels, in the case of ethanol, and many biochemicals and biopolymers of varied industrial application [2]. Microorganisms (bacteria, yeasts, fungi, and microalgae) can accumulate over 20% of their cell mass as lipids, deeming them oilseeds because of their propensity to accumulate over 20–25% (*w*/*w*) of lipids. Deficiencies in nutrients, such as nitrogen or phosphorus, or by excess content of carbon can lead to the accumulation of cell storage lipids in these microbes. Normal cellular functions like nucleic acid and protein synthesis occur under these conditions, and eventually, cell growth ceases, causing the oilseed species to consume any carbon source available and convert it to lipids through biochemical reactions [3].

Single-celled oils (SCOs) come from microbial sources; they are basically microbial oils, with a composition remarkably like that of edible plant oils, animal oils and fats.

The potential of these oils and fats of microbial origin has been recognized since the last century. Before 1980 some scholars focused their study on the biochemistry and metabolism of these oleaginous microorganisms in the synthesis of these oils. In the last 20 years, the production of SCO and its oily compounds has become one of the most popular branches of research in this field, as the products obtained from SCO have been shown to play a critical role in human health [4].

Such microorganisms can produce oily products, are adaptable and grow on different carbon sources, consume volatile fatty acids, and have long-chain fatty acid profiles. Bettencourt et al. [5] studied four yeasts isolated from the aforementioned species: *Apiotrichum brassicae*, *Candida tropicalis*, *Metschnikowia pulcherrima*, and *Pichia kudriavzevii*. The culture medium was supplemented with seven different volatile fatty acids. The findings demonstrated that both in a synthetic medium and in the waste-derived effluent filtrate, the yeasts *A. brassicae* and *P. kudriavzevii* had a productivity of 40% (*w*/*w*), with palmitic acid (20%) and stearic acid (20%) lipids being the most prevalent lipids (>40%). Utilizing other carbon sources and employing these isolates, there is a great potential for producing SCO on a wider scale, combining economic and environmental advantages [5].

*Arundo donax* L., a prospective source of carbohydrates that can be converted to SCO by oleaginous yeasts, was examined by Di Fidio et al. [6]. These yeasts can transform the cellulose and hemicellulose fractions into oils, which is a crucial step in improving the economic viability of SCO production. These authors used two xylose hydrolysates produced by pretreatment (microwave-assisted hydrolysis of hemicellulose catalyzed by FeCl3 or Amberlyst-70) and two glucose hydrolysates produced by enzymatic cellulose hydrolysis to develop the bacterium *Lipomyces starkeyi* DSM 70.296. With a yield of 30% by weight, the bacterium *L. starkeyi* was successfully grown in non-detoxified and partially-detoxified hydrolysates. This study showed that lignocellulosic raw material from perennial grasses could be efficiently used to create next generation biodiesel and high added value products, with integrated cascade hydrolysis processes being able to produce SCO.

### 1.2. Second-Generation Biorefineries

When discussing second generation biorefineries, we take into account those that employ biomass, such as grasses grown in arid circumstances, agroforestry leftovers, and any sort of harvest residue. First generation biorefineries use inputs that are collected directly from sugar and starch [7].

Adding value to lignocellulosic materials is possible with a biorefinery, which has sparked extensive study into how to transform their cellulose, hemicellulose, and lignin constituents into goods with a high added value. However, because of their strong resistance to enzymatic and chemical hydrolysis, such materials must first undergo a pre-treatment that separates the cellulose/hemicellulose and lignin fractions [6,7].

According to Huang et al. [8], numerous initiatives have concentrated on the use of inexpensive materials as a method of creating SCO in order to employ biomass in the manufacture of microbial oil. The most common methods of lipid synthesis in oleaginous microorganisms are “de novo” and “ex novo” lipid accumulation methods. The first method, which utilizes hydrophiles, typically necessitates nitrogen-limited growing conditions. Additionally, “ex novo” lipid synthesis involves the fermentation of SCO in hydrophobic substrates.

The same authors mentioned above claim that there are two drawbacks to low-cost substrates that can reduce the cost of SCO manufacture and can obstruct the industrialization process. The first would be that there are issues with the transportation of raw materials, and the second would be that there are limitations in the availability of raw materials, which is negative for more steady SCO production. Consequently, the utilization of lignocelluosic biomass, the most accessible and sustainable source of SCO in nature, might be extremely intriguing for the clean and sustainable production of SCO.

Although the utilization of lignocellulosic biomass to make microbial oil is still in its infancy, it is extremely well-known in the production of second-generation bioethanol. The BM2BD (biomass for biodiesel) plan, which concentrates on lignocellulosic biomass for biodiesel production, is carried out in three stages: in the first stage, the lignocellulosic biomass is converted into fermentable sugars; in the second stage, these sugars can be converted into microbial lipids by oleaginous microorganisms; and in the third and final stage, the microbial lipids are converted into biodiesel [8,9].

Liang et al. [10] performed the pre-treatment of sweet sorghum bagasse at 100 °C using lime at concentrations of 0, 0.05 and 0.1 per gram of bagasse for 1 or 2 h. When analyzing the composition of the pretreated samples, they revealed that lignin and xylan were more easily removed with increasing lime concentrations. The combined effect was evaluated relating pretreatment and enzymatic hydrolysis. Under the condition of 0.1 g ATR/g bagasse, 10 mL water/g bagasse and 2 h of treatment, it was possible to obtain a sugar yield of 73.6%. The sludge was used as a substrate for the growth of *Cryptococcus curvatus*, an oleaginous yeast. The yield of neutral lipids (g neutral lipid/g sugar) obtained was 0.19, close to the theoretical value. Thus, it is possible to convert sorghum bagasse into adequate lipids to produce biodiesel, thus valuing an agricultural by-product for the generation of renewable biodiesel raw materials. Numerous studies have concentrated on selecting the best strains to perform lipid synthesis from lignocellulosic hydrolysates, according to Huang et al. [8]. The oleaginous microbes *Yarrowia lipolytica*, *Lipomyces starkeyi*, *Rhodotorula glutinis*, and *Rhodosporidium toruloides* have all been reported to be able to utilize lignocellulosic substrates to generate SCO.

The main difference between a second-generation biorefinery and an SCO (single cell oil) lignocellulosic biorefinery would be the use of biomass as raw material and in oil production. While the second-generation oil biorefinery uses biomass to produce fermentable sugars, later converted into oils by yeast, the SCO lignocellulosic biorefinery uses microorganisms directly to produce oils [3,4].

Integrating these two types of biorefinery is possible and may allow more efficient use of biomass and the production of a broader range of renewable products, contributing to sustainability and reducing greenhouse gas emissions. The primary possibility for integration would be to use lignocellulosic biomass as a source of fermentable sugars to feed the microorganisms used in the production of oils by the SCO lignocellulosic biorefinery. The oils produced are refined and used as raw material in the production of liquid biofuels by the second-generation oil biorefinery. This can be done, for example, by using oils rich in long-chain fatty acids, produced from microalgae, as the raw material for biodiesel production. The other possibility is to use the waste from the production of oils by the SCO lignocellulosic biorefinery as a source of biomass to feed the second-generation oil biorefinery. These residues can be used as a source of cellulose and hemicellulose to produce fermentable sugars, which are then converted into oils by yeasts in the second-generation oil biorefinery.

### 1.3. Potential Markets for Microbial Lipids

Economic factors are crucial to a SCO biorefinery’s performance, according to Jin et al. [11]. The utilization of heterotrophic oleaginous bacteria to manufacture SCO is the subject of very little techno-economic research at the moment. Ratledge and Cohen [12] stated an expected price for microbial oil of USD3000/tonne, this without the cost of raw materials and based on work done in New Zealand to produce the equivalent of cocoa butter from lactose using *Candida curvata*. In Table 1 it is possible to observe some yeast strains with the capacity to produce lipids, some of which involve the use of lignocellulosic hydrolysates.

We have seen from the above that many microorganisms can accumulate lipids as intracellular storage compounds, such as yeasts, fungi, molds, and microalgae. SCOs form lipid droplets that can be found intracellularly or in algal chloroplasts. Fatty acid analysis was done, and revealed that they were similar to those found in vegetable oils (most used to produce first-generation biodiesel). Additionally, the polyunsaturated fatty acids (PUFAs), also known as essential fatty acids, which cannot be produced by mammals and must be acquired from the diet, are present in large amounts in the fatty acid profile from SCO. Therefore, if these PUFAs were employed as a dietary supplement, there would be more accessibility for the population [25].

## 2. SCO Microbes and Culture Conditions

Triacylglycerols and free fatty acids make up the majority of the lipids generated by oleaginous organisms. Due to their high carbon-to-heteroatom ratios, lipids are desirable raw materials for the manufacture of renewable fuels [11]. The industrial application of microbial lipids may be limited by the high cost in the fermentation process, mainly due to the carbon source, to obtain SCO. Therefore, much research has been directed towards the efficient use of organic waste as a carbon source for microbial lipids, such as lignocellulosic biomass, which would definitely increase the profitability of the production process and boost a biologically-based economy [26].

Lipid-producing oleaginous microorganisms, according to Ma et al. [27], can be classified into three groups: microalgae, fungi (mold and yeast) and bacteria. Bacteria are capable of producing fewer lipids, as they can only synthesize specific lipids and PUFA (polyunsaturated fatty acids), which makes microalgae and fungi the main producers of lipids to be used in biorefineries. However, a microorganism is only considered oleaginous when it is able to accumulate more than 20% of its dry body mass as oil in the form of lipids [10]. According to Ma et al. [27] for holophytic microalgae, the best indicator of total lipid production capacity is productivity, which takes into account biomass productivity and lipid content, rather than just the lipid content produced.

Ageitos et al. [28] state that oleaginous yeasts may collect lipids in amounts ranging from 20% to 80% of their dry cell weight, with just 5% of them being able to accumulate lipids in amounts of more than 25%. *Yarrowia*, *Candida*, *Rhodotorula*, *Rhodosporidium*, *Cryptococcus*, *Trichosporon*, and Lipomyces are some of the genera of oilseed yeast. For instance, 58%, 65%, 64%, and 72% of lipids are produced by *Candida curvata*, *Cryptococcus albidus*, *Lipomyces starkeyi*, and *Rhodotorula glutinis*, respectively [29]. When compared to other microbial sources, yeasts offer several benefits for the generation of lipids. Their cultures may be expanded more readily than those of microalgae, and their doubling times are often less than one hour. Additionally, oily yeasts can be helpful for the generation of triglycerides, surfactants, or polyunsaturated fatty acids due to the variety of microorganisms and growth conditions [28].

In the presence of sunshine and carbon dioxide from flue gases, microalgae are unicellular photosynthetic organisms that are able to produce significant quantities of lipids and hydrocarbons. Lipid production from algal cells ranges in average content from 1 to 70%, but under some circumstances, it may reach 90% of the dry weight. *Botryococcus braunii* (25–75%), *Chlorella* sp. (28–32%), *Nannochloropsis* sp. (31–68%), *Nitzschia* sp. (45–47%), and *Schizochytrium* sp. (50–77%), among others, are microalgae that generate lipids [29].

In comparison to land plants and other microorganisms, microalgae have a number of advantages, including high lipid productivity, four distinct cultivation modes (autotrophic, heterotrophic, mixotrophic, and photo-heterotrophic), independence from external environmental conditions or arable land, efficient nutrient extraction, and a high growth rate [30]. Heterotrophic microalgae culture is often cheaper, easier to set up, and easier than autotrophic cultivation among crops, enabling large-scale applications including combined or separate wastewater treatment and biofuel generation [31]. Through the use of sufficient carbon concentrations and alternative or renewable sources, such as lignocellulosic biomass from agricultural or forestry residues, or other substrates of organic residues, yields from microorganisms must be increased and made more affordable in order to produce lipids [32]. Since it is made up of cellulose, hemicellulose, and lignin, non-edible lignocellulosic biomass is the most prevalent renewable source in the biosphere. It has been widely employed as a raw material in the manufacturing of biofuels to hydrolyze diverse inedible lignocellulosic biomass to produce fermentable sugars, whether by physical, chemical, or enzymatic means [33]. A potential approach to obtaining the commercial viability of microbial lipids is when it is coupled with cultures of oleaginous bacteria. But each microorganism’s and each substrate’s features must be taken into account at the same time in order to achieve an efficient bioconversion of inexpensive substrates into microbial lipids [32]. It is not possible to describe the carbon concentrations necessary for good productivity due to the great heterogeneity of the action of the yeasts and the lignocellulosic raw materials used, in addition to the conditions of the applied processes, such as time and temperature parameters and whether or not pre-treatments are carried out. However, according to Mu et al. [34] it is possible to consider the process carried out with the biomass concentration of 5.8 g/L and the produced lipid content of 34.0%, with the use of the *Chlorella* sp., as a good productivity result. Oleaginous bacteria have the unusual capacity to absorb several carbon sources from renewable substrates, including hydrophilic and hydrophobic, claim Patel et al. [30]. Sugarcane wastes are one of the best raw material options to be used in the production of such microorganisms and lipids. After the enzymatic hydrolysis of this material, it is possible to obtain a hydrolysate rich in glucose, xylose, and arabinose and this substrate can be used by microorganisms such as *Chlorella* sp. and the production of lipids [34].

Ex novo lipid synthesis is more advantageous for growth on hydrophobic substrates, but it yields fewer triacylglycerols than de novo lipid synthesis on sugar-based substrates. These substrates build up in oleaginous bacteria through three distinct physiological phases: growth, lipid accumulation, and lipid turnover when there is a high carbon/nitrogen ratio present. During the growth phase, available nutrients, particularly the carbon source, are taken up to create biomass, proteins, polysaccharides, and polar lipids. Wynn et al. [35] state that this lipid accumulation takes place during the stationary phase under conditions of surplus carbon and nutritional constraint, which prevent cell growth and division because of a shortage of nitrogen. The extra carbon is then converted into storage lipids. In addition to nutrients, temperature, pH, aeration, light, and CO_2_ levels all affect the formation of lipids.

Understanding the effects of biomass inhibitors on the performance of microbial strains is crucial for the development of methods that can lead to increased fermentation performance, according to Liu et al. [33]. Toxic compounds like furfural, vanillin, and 5-hydroxymethylfurfural (5-HMF) are present in lignocellulosic hydrolysates and are one of the major issues affecting the effectiveness of fermentation processes based on hydrolysates. As a result, they draw attention to this issue and suggest using evolved strains as a potential solution to obtain oleaginous microorganisms with improved capacity to tolerate toxic compounds and grow in biomass hydrolysates. According to Liu et al. [36] the inhibitory concentrations will vary according to each microorganism, however values between 4–8 mM for furfural, 0.5–3 mM for vanillin and 1–5 mM for hydroxymethylfurfural can be found for a wide variety of microorganisms, based on tests performed with *R. toruloides*. By comparing oilseed yeast strains from five different genera, Ngamsirisomsakul et al. [37] were able to determine which strain would be best for using sugarcane bagasse as a raw material for the manufacture of lipids. It is possible that sugarcane bagasse can be used as a raw material for the production of microbial lipids if the control criteria are met because the hydrolysate’s buffering capacity improved the growth and production of lipids in all strains, showing better performance in the pH range between 5.5 and 7.5, temperature of 30 °C, and the use of a flask shaking at a speed of 200 rpm.

Numerous microalgal strains have the ability to use organic acids, sugars, and alcohols as carbon sources when grown under heterotrophic or mixotrophic conditions. The difference is that during heterotrophic development, microalgae are grown in the dark, ingest organic materials from their surroundings to meet their energy needs, and emit CO_2_. Microalgae in a mixotrophic culture use light energy to consume CO_2_ as well as exogenous organic substances from the environment [38]. The high quantity of cellular lipids in algae depends on the stage of cell growth and is typically attained under environmental stress, which can be brought on by restrictions in nitrogen, phosphorus, silicon, salinity, and iron, according to Subramaniam et al. [29]. The growth, lipid accumulation, and chrysolamine of *T. utriculosum* were studied by Wang et al. [39]. The results showed that the highest biomass concentration (6.21 ± 0.17 g/L) was obtained at 3 mM of initial nitrogen concentration, whereas higher initial nitrogen concentrations, such as 18 mM, were more prone to lipid accumulation and chrysolamine accumulation, leading to higher lipid content (43.80 ± 0.57% of DW). The ability of *Tetradesmus obliquus SGM19*, freshwater microalgae belonging to the Scenedesmus genus, to produce lipids and beta-carotene was investigated by Singh et al. [40]. The strain demonstrated ideal growth circumstances with a nitrogen supply of 1.5 g/L of NaNO3, 12L:12D photoperiod, obtaining 2.5 g/L of biomass, 0.67 mg/g of β-carotene in dry biomass, and 28.5% lipids by weight, where the profile was made of 71% C16 and C18 fatty acids. As determined by the lipid content, the biodiesel’s characteristics (cetane number = 48.59) fell within the ASTM D6751 and EN 14,214 standards’ tolerances. The type and concentration of the nitrogen source can affect the growth rate, lipid content, and fatty acid composition of microbial cells. Generally, using organic nitrogen sources such as yeast extract, peptone, or tryptone has resulted in higher biomass and lipid yields than inorganic nitrogen sources such as ammonium sulfate or nitrate [41,42].

Among the main factors reported that interfere with the intracellular accumulation of lipids in microorganisms are: (1) Carbon source—in general, quickly-metabolized carbon sources, such as glucose, can lead to high biomass yields but can result in lower accumulation of lipids compared to more complex or less quickly-metabolized carbon sources, such as xylose or cellobiose, which allow more significant accumulation of lipids [43]. (2) Oxygen availability, since insufficient oxygen supply can limit the growth of microorganisms and reduce lipid accumulation [44]. (3) Temperature, as lower temperatures can result in a more significant accumulation of lipids, while higher temperatures can lead to faster growth but less accumulation of lipids [45]. (4) The pH has a significant influence since different microorganisms have different ideal pH ranges for growth and lipid accumulation [43,44,45]. (5) The availability of nutrients because, in addition to nitrogen, the presence of phosphorus, sulfur, and trace elements can also affect the accumulation of lipids, and the insufficient availability of these nutrients can limit the growth and accumulation of lipids in microorganisms [46]. The use of symbiotic cultures (mixed or sequential) has several advantages over pure cultures and can be a strategy to increase the production of microbial lipids and carotenoids. When used in conjunction with low-cost substrates like industrial waste or effluent, processing costs can be decreased. Dias et al. [47] report that some species of yeast and microalgae have comprehensive nutritional needs. One of the most promising methods for producing biodiesel and oleochemicals is the synthesis of microbial lipids from renewable raw sources. Investments in a mix of metabolic systems engineering, multi-omics integration analysis, computer-aided designs, and synthetic biology methodologies are required to improve the production/accumulation of lipids by microorganisms relative to the theoretical maximum limitations [48].

**Figure 1:**
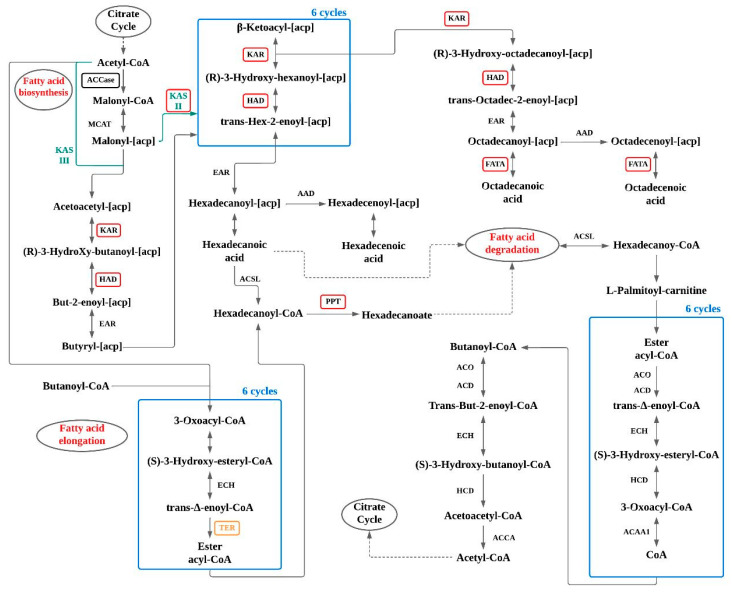
Schematic diagram of fatty acid metabolic pathways, adapted from [49].

In Figure 1 it is possible to observe the schematic diagram of fatty acid metabolic pathways.

## 3. SCO Production from Lignocellulosic Biomass

### 3.1. Stages of the Bioconversion of Lignocellulosic Biomass to Lipid

**Figure 2:**
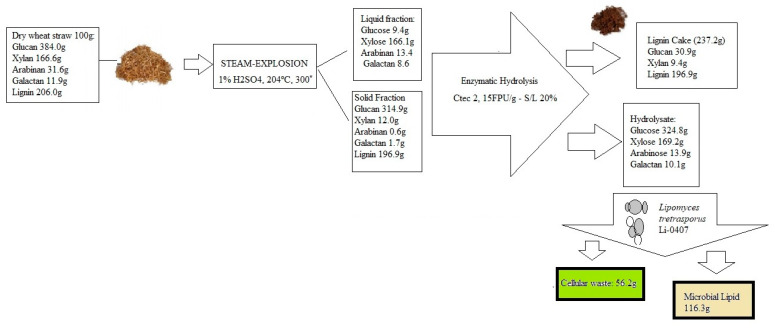
Microbial lipid production from wheat straw using the oleaginous yeasts *L. tetrasporus* DSM 70314, adapted from [75].

The success of the conversion of lignocellulosic biomass to lipid from oleaginous microorganisms is dependent on specific pretreatments such as acid, alkaline, sequential acid-alkaline, steam explosion, and organosolv pretreatment [50]. Pretreatment allows the disruption of the lignocellulosic matrix to deliver the sugars consumed during the microbial lipid fermentation [8,51,52,53,54,55,56]. Acid pretreatments are well documented in the literature as the most applied method for high sugar yield from a lignocellulosic matrix [57] and the most commonly used is sulfuric acid [50,58]. Alkaline pretreatment is another chemical process widely used to improve the enzymatic hydrolysis of several lignocellulosic matrices [58], which is highly effective in lignin solubilization [59], and less aggressive with sugars [60]. The sequential acid-alkaline pretreatment is also an efficient alternative for processing lignocellulosic biomass. The main advantage of this combined process is the effective extraction of hemicellulose and lignin through the acid and alkaline stages, respectively, and a reduction in byproducts [61]. However, the number of stages, processing time, and investment in equipment required are considered the main disadvantages [62]. In addition, an essential group of pretreatments has gained significant importance. They are those carried out with ionic liquids, which can be considered more environmentally friendly. They include a wide variety of chemical components, with the process generally carried out in milder temperatures or environments, as is the case with the use of choline acetate [63] and 1-butyl-3-methylimidazolium chloride ([BMIM]CL) [64]. The application of high pressure and high temperature for a short time, namely steam explosion, is frequently used to extract lignocellulosic biomass from hardwoods and herbs [65]. In addition, several organic solvents (e.g., methanol, ethanol, acetic acid, formic acid, acetone, phenol, or glycerol) are frequently used to separate lignin (liquid fraction) and cellulose (solid fraction) from lignocellulosic biomass [66,67]. The pretreated biomass is mixed with different types of enzymes (e.g., cellulases and hemicellulases) for the enzymatic hydrolysis process of polysaccharides into monosaccharides (e.g., glucose and xylose) [68]. Enzymatic hydrolysis can be performed with lipid fermentation in two processes called separate hydrolysis and fermentation (SHF) and simultaneous saccharification and fermentation (SSF) [11]. The use of the SSF method is preferable since its application reduces the risk of enzyme inhibition by sugar accumulation, cross-contamination of the fermentation process, processing time, and economic costs [69,70,71]. The temperature of the SSF process is an important parameter to consider since enzymatic hydrolysis and fermentation occur at different temperatures [72,73,74]. The use of a thermotolerant fermenting microorganism should be considered as a good strategy to optimize the SSF process since enzymes are more sensitive to temperature variations than thermotolerant microbes. It is possible to observe in Figure 2 an example of a SCO production process from dry wheat straw wastes.

### 3.2. SCO Production from Lignocellulosic Biomass and Setbacks

The production of SCO from lignocellulosic-hydrolysates biomass can be performed by fungi species (yeasts and filamentous fungi), bacteria, and microalgae [53,55,76,77,78,79,80,81,82]. Yeasts are the main microorganisms used in the processes of converting lignocellulosic biomass into lipids [50]. Recently, the use of corncobs as the substrate to produce SCO from *Rhodotorula taiwanensis* (AM2353) has achieved a yield of 55.8 g of oil per kilogram of corncobs and more than 81.5% of the oil could be converted into biodiesel [55]. An innovative source of biomass is the native grass from the *Cyperus distans* species, frequently available in roadsides, wetlands, and abandoned areas [53]. Its use was recently published as a promising substrate for SCO production. Using *Yarrowia lipolytica* (MTCC 9519) in an SCO biorefinery it was possible to reach about 53.62% of lipid accumulation in the cells [53]. Rice straw is widely used as biomass and is another source for SCO production. A recent study investigated the performance of *Cryptococcus curvatus* (ATCC 20509) for lipid fermentation [80]. The results of this study showed that its hydrolysates were able to produce a high total lipid of 8.8 g/L with 0.17 g/g of lipid yield from the present substrate [80]. The use of sugarcane bagasse and rice husk was also evaluated for the production of biodiesel by six yeast isolates [*Meyerozyma guilliermondii* (G5 MK414782), *Pichia kudriavzevii* (G9 MH000699), *Pichia manshurica* (G10 MH279643), *Pichia kudriavzevii* (SY2 MF926445), *Candida albicans* (SY3 MG996750), and *Rhodotorula mucilaginosa* (SY4 MH279637)] [83]. In this study, all lignocellulosic biomass was pretreated by steam explosion alone to avoid contamination with hydroxymethylfurfural (HMF), furfural, and acetic acid. The use of sugarcane bagasse hydrolysate as a substrate achieved maximum lipid content (37.99%) from the strain *Meyerozyma guilliermondii* (G5 MK414782) and higher lipid accumulation (2.39 g/L) was obtained from the strain *Pichia kudriavzevii* (SY2 MF926445) when the substrate used was rice husk hydrolysate [83]. For the manufacture of SCO, corn stover is a fantastic source of lignocellulosic biomass. Its use was recently carried out through lipid fermentation using the strain *Trichosporon dermatis* 32903, which was chosen from eleven oleaginous yeast strains: *T. mucoides* (1367), *T. mucoides* (1368), *T. dermatis* (32856), *Yarrowia lipolytica* (31596), *Rhodotorula mucilaginosa* (31029), *Candida utilis* (1768), *T. coremiiforme* (1256), *Rhodosporidium toruloides* (NP-11), and *Y. lipolytica* (XYL+) [84]. The strain T. dermatis (32903) presented 7.46 g/L (0.104 g/g of lipid yield) and 6.81 g/L (0.101 g/g of lipid yield) from dilute-acid and dilute-alkali pretreated corn stover, respectively. The strategy to wash the pretreated corn stover has proved to be essential in improving lipid production and sugar to lipid yields by removing the contaminants or inhibitors. As result, the strain *T. dermatis* (32903) showed higher 11.43 g/L (0.156 g/g lipid yield) and 20.36 g/L (0.186 g/g lipid yield) lipid production from washed dilute-acid and washed dilute-alkali pretreated corn stover, respectively [84]. The potential of SCO production from sweet sorghum stalks was evaluated in the presence of the yeast *Lipomyces starkeyi* (CBS 1807) [85]. Using the hydrolysate sweet sorghum stalk juice with an initial sorghum content of 12% (*w*/*w*), the highest lipid yield of 6.40 g/L was obtained with lipid content of 29.5% (*w*/*w*) [85]. The use of oleaginous bacteria species, mainly *Rhodococcus opacus*, has been frequently tested for its SCO production from lignocellulosic biomass [76,77,81]. From kraft hardwood pulp a lipid content was achieved of 46% *w*/*w* with *R. opacus* (PD630) [77]. Another study explored the use of oxygen-pretreated kraft lignin from black liquor followed by fermentation with *R. opacus* (DSM 1069) which presented a maximum lipid yield of 0.067 mg/mL with palmitic (46.9%) and stearic (42.7%) acids being the principal lipid products [81]. The use of both pretreated lignin with ethanol and ultrasound has demonstrated suitable lipid production capacity (4.08%) with *R. opacus* (DSM 1069) [81]. The same bacterium strain *R. opacus* (DSM 1069) was responsible for the bioconversion of lignocellulosic pretreatment effluent [82]. The lipid accumulation in the cells was about 26.99% of its cellular dry weight and represents a novel biodiesel production and waste treatment route [82]. The use of forest biomass (Norway spruce and silver birch) as a substrate for sustainable SCO production via oleaginous green microalgae, especially the species *Auxenochlorella protothecoides*, has been established [79]. When the microalgae were cultured on birch and spruce substrate hydrolysates, respectively, and the forest biomass was processed using a hybrid organosolv-steam explosion approach, lipid production of 5.65 g/L (66% lipid content) and 5.28 g/L (63.08% lipid content) was produced [79].

The C:N ratio of the hydrolysates, sugar content, sugar combinations, and the presence of impurities and inhibitors from the necessary pretreatment stage of the process are only a few difficult obstacles that have a direct influence on the yield of SCO production [11]. The primary obstacle that hinders the growth of the oleaginous microbe and SCO formation is the synthesis of inhibitory chemicals, such as HMF, furfural, acetic acid, neutral and acidic phenolics, and other chemical species [36,86,87]. Some strategies have been adopted to face such setbacks such as the use of surfactants, the inclusion of detoxification processes, and genetically modified strains with resistance/tolerance to the inhibitor compounds. Despite the ability of some microorganisms to accumulate lipid in their cells in the presence of inhibitors, the process of detoxification is crucial to enhance microbial lipid production but incurs additional economic cost and energy use [88]. In a recent study using giant reeds (*Arundo donax* L.) as a promising substrate for SCO by oleaginous yeast (*Lipomyces starkeyi* DSM 70296), the authors found 8 g SCO from 100 g biomass with a lipid yield range of 15–24 wt% [6]. The use of barley hull hydrolysate was assessed for SCO production by the *Trichosporon cutaneum* (CTM-30125) strain. The process of detoxification with overliming and adsorption treatments before fermentation was essential to enhance the accumulation of microbial lipid in the cells of *T. cutaneum* (CTM-30125) [89]. Corn stover was used for the production of SCO via *T. cutaneum* CX1 under the evaluation of two principal technical setbacks (Inhibitor contaminants and low C/N ratio) [86]. The authors tested a biodetoxification method and the process was stopped when HMF and furfural compounds were undetectable. The applied method contributes directly to the inhibitor removal and increase of C/N ration with the reduction in nitrogen content. According to the authors, the bioaccumulation of lipid by *T. cutaneum* CX1 using the bio-detoxified hydrolysate reached 23.5%, 2 times more than the non-detoxified substrate [86]. Despite the economic and energy increase related to the detoxification process, mostly recent studies indicate that the development of detoxification strategies aimed at high yield lipid content for SCO production is essential. It is possible to observe a comparison of SCO production by different microorganisms in Table 2.

### 3.3. Strain Development

Several oleaginous microorganisms have been screened in lipid production, including microalgae, bacteria, filamentous fungi, and yeasts [109]. These microorganisms share a common feature in their metabolism, which is adapted to convert carbon substrate into storage lipids, accumulated in cytoplasm, mainly in the form of triacylglycerides (TAG, referred to single-cell oils—SCO) and free fatty acids (FFA) [50]. Oleaginous microorganisms designated as valuable candidates for lipid biosynthesis present: (i) fast growth rates, (ii) high intracellular lipid accumulation and (iii) the ability to grow in low-cost media, such as agro-industrial wastes or lignocellulosic materials [110,111,112]. In particularly, a variety of factors, typically species-specific, has an impact on the capacity of oleaginous bacteria to collect lipids. However, nutritional imbalance, such as nitrogen famine conditions (or sulfate, phosphate, or magnesium deficiency) in the presence of abundant carbon, is a frequent cause of oil buildup. This condition impairs cell proliferation and increases lipid biosynthesis. Other physical parameters also affect the accumulation of lipids, such as light intensity, temperature, pH, dissolved oxygen and culture agitation [113,114,115]. Metabolic regulation in oleaginous microorganisms is used as a biological tool to develop metabolic engineering strains to increase lipid production and develop robust strains that have other desired characteristics, such as survival under adverse conditions, extension of the substrate spectrum, and favoring downstream processing to lipid recovery [77,110,116,117,118].

Metabolic engineering methods include random mutagenesis or targeted genes, by gene knockouts or overexpression. Random mutagenesis is carried out by physical or chemical treatments, which induce damage to the microorganism’s DNA, followed by mutant selection. These mutagen treatments include radiation (e.g., ultraviolet and gamma radiation), low energy nitrogen ions, and chemical agents (e.g., formaldehyde and ethidium bromide) [110,111]. However, random mutagenesis generates several mutants, which makes the step of mutant selection time-consuming. The development of a targeted genetic engineering microorganism is addressed to gene deletion, gene overexpression or gene introduction. The deletion of target genes, responsible for β-oxidation (e.g., acyl-CoA oxidases, and lipases *TGL4* and *PEX10*), inhibits lipid degradation. In contrast, the overexpression of diacylglycerol O-acyltransferases *DGA1* and acetyl-CoA carboxylase *ACC1* genes improves lipid accumulation [111,112,119]. The overexpression of malic enzymes in engineered *Rhodotorula mucilahodotorula* provided a NADPH cofactor for fatty acid biosynthesis and increased SCO production by 18% compared to wild strains and resulted in improved lipid quality [116]. More than improvement of lipid accumulation, advances in genetic knowledge have enabled the production of modified fatty acids with high value-added applications [112]. Engineered microorganisms, such as *Rhodococcus opacus,* are also capable of utilizing high concentrations of xylose for SCO production, achieved by the introduction of two heterologous genes from *Streptomyces* encoding xylose isomerase (*xylA*) and xylulokinase (*xylB*) [77]. In Table 3 it is possible to verify the main strategies used for the development of strains.

### 3.4. Lipid Recovery

**Figure 3:**
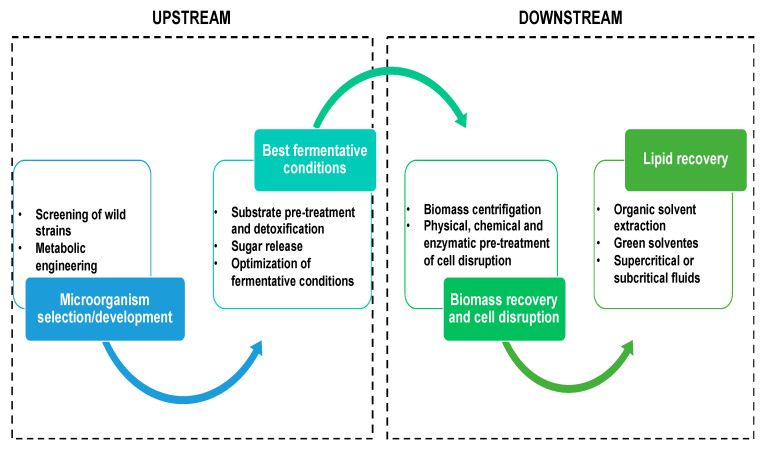
Upstream and downstream steps of SCO production from lignocellulosic biomass.

In the upstream steps of SCO production, the best fermentative conditions are performed to reach the maximum microbial performance in lipid production, e.g., optimization of fermentative parameters and strain development by genetic modifications. The downstream process also presents critical steps that affect process efficiency, which include methods for lipid recovery from oleaginous microorganisms, such as cell disruption pre-treatments, and solvent and/or assisted extraction techniques [124,125]. Due to the rigidity of microbial cell walls, compared to other biological systems (plant and animal cells), the use of conventional solvent maceration alone results in incomplete extraction. Thus, physical (mechanical and non-mechanical), chemical and enzymatic methods of cell disruption have been studied to increase lipid recovery (Figure 3) [44,109,126]. Although all of these methods of microbial cell disruption are described in the literature, physical disruption procedures have been more relevant for large-scale applications, e.g., bead milling, high-pressure homogenization, microwave and ultrasound-assisted extraction methods.

After fermentation, a thermal treatment (pasteurization) is usually performed to inactivate enzymes (e.g., lipases) and prevent the microorganism’s consumption of storage lipids. Then, the biomass is concentrated by centrifugation and to the next step is cell disruption. SCO present similar oxidation and rancidity issues to plant seed oils, due to their polyunsaturated-fatty-acids content. Thus, the entire procedure of lipid recovery requires mild temperatures to prevent lipid damage. In particular, mechanical methods of cell disruption results in temperature rise, making it necessary to link cooling systems to these procedures [12,44,124,127].

Among mechanical methods of cell disruption, the bead-milling technique is simple and one of the most efficient, suitable for cells of yeast, bacteria, algae, and filamentous fungi. This method disrupts cells by the abrasive action of small beads of glass or stainless steel against cells to grind the oleaginous microorganisms and release lipids. The optimal diameter of the beads is 0.5 mm for all microorganism cells, except for bacteria, where small beads (0.1 mm) improve the efficiency of cell disruption. At the end of the process, the beads are easily removed from the solution by gravity and lipid recovery is subsequently performed by solvent extraction [44,127,128,129,130,131,132,133,134,135,136,137,138,139]. A combined enzymatic method of cell disruption by bead milling, applying lipase, phospholipase, protease, and cellulase, increased lipid recovery by more than 40%. The extraction of SCO from the microalgae *Chlorella vulgaris* treated by bead milling resulted in a lipid yield of 75%, while by combining enzymatic hydrolysis to bead milling yielded 88% of lipids [140]. Comparing bead milling to microwave and ultrasound methods for lipid extraction from the yeast *Yarrowia lipolytica*, bead milling proved to be the most efficient and low-energy-consuming technique, causing no degradation of fatty acids or lipid profile modification [138].

The method of high-pressure homogenization has been widely applied in the dairy and fruit-based processing industries. This technique is also applied to cell disruption in lipid extraction, which is achieved by forcing the suspension of cells at high pressure through a small gap, reaching up to 150–200 MPa. The main advantage of high-pressure homogenization is its easy scalability [44,124,126,127]. This method is suitable for all microorganisms’ cells, except for filamentous fungi, which can block the homogenization valve by mycelium structures, but low biomass concentrations may prevent this limitation [26,44]. Including the pretreatment of high-pressure homogenization to *Y. lipolytica* cultures increases oil-extraction yield in four times, compared to only-solvent maceration [106,141]. Some authors have also suggested that combined pretreatments of bead milling and high-pressure homogenization also maximize lipid extraction from *Y. lipolytica* cultures and decrease energy consumption [142].

Among non-mechanical pretreatments of cell disruption, microwave technology is safe, inexpensive, and effective. Cell disruption is achieved by microwaves (ranging between 0.3 to 300 GHz), which interact with free water molecules inside the cells, resulting in temperature rise and cell lysis [44]. Microwave pretreatment in SCO extraction increases oil yields and quality, and decreases extraction time. The maximum oil yield from the green algae *Scenedesmus obliquus* was achieved after 30 min of microwave treatment, yielding 77% of total recoverable oil [133]. Furthermore, the association of microwave pre-treatment to enzymatic hydrolysis increased SCO extraction, obtaining almost 97% of total lipids from the culture of *Rhodosporidium toruloides* [134]. Another non-mechanical pre-treatment is ultrasonic-assisted extraction, which is based on cavitational phenomena, generated by an intense sound wave (20 kHz to 10 MHz), which results in the collapse of gas bubbles, liquid shear forces and cell disruption [142]. The combined pretreatments of ultrasound and microwaves increased extraction efficiency and quality (fatty acids composition) of SCO from *Mortierella isabellina,* yielding almost 93% of lipids [135]. However, the disadvantage of both pretreatment methods is temperature rise, which in some contexts could modify the chemical profile of lipids, affecting product quality [44].

In SCO recovery, cell disruption pretreatments are followed by organic solvent extraction, mainly developed by Soxhlet, Bligh and Dyer, or Folch standard extraction methods [136]. The Soxhlet method is applied to solid or dried ground samples, extracted by several washing cycles with organic solvents. In this method, the high temperature of organic solvents under reflux makes this technique unsuitable for SCO containing unsaturated fatty acids to avoid damage to the lipids [44]. The Bligh and Dyer method is carried out with a chloroform-methanol system, in which lipid fraction occurs in the chloroform phase and hydrophilic substances are removed in the methanol and water phase. The Folch method is similar to Bligh and Dyer, but differs in solvent volumetric ratios and in the washing procedure with a salt solution [26]. Despite the high extraction efficiency of chloroform, methanol and hexane, studies have been carried out to replace toxic and harmful solvents with environmentally friendly solvents, aiming to benefit the oil industry sector. At laboratory scale, hexane was replaced by isoamyl acetate, which increased SCO extraction from *Y. lipolytica*, pretreated with bead milling and high-pressure homogenization [123]. Another strategy to replace toxic solvents is the use of supercritical or subcritical fluids, such as carbon dioxide, established as the most common solvent for lipid and hydrophobic compound extraction, causing no contamination of SCO with toxic solvents or thermal degradation [44,126]. Although organic solvent use affords superior lipid extraction yields compared to supercritical fluids, some authors demonstrated that carbon dioxide simplifies oil refinement steps and excludes the solvent distillation stage [137]. Thus, studies need to optimize parameters such as supercritical fluid, temperature, pressure and cell disruption pretreatment to increase lipid yield in supercritical extraction, and expand this emerging technology use in SCO extraction.

## 4. Microbes, Feedstocks and Coproducts for Single-Cell-Oil Biorefineries

Single cell oils (SCO), also known as glyceride oils, are produced by oleaginous microorganisms such as bacteria, yeast, fungus, and microalgae [138]. The intracellular lipid content of these oleaginous bacteria can increase to as much as 60 to 70 percent of weight in some species, or around 40% of the dry cell weight [139].

Lipid production is facilitated by oleaginous bacteria. The *Arthrobacter* species, *Gordonia* species, *Acinetobacter* species, and *Rhodococcus* species are a few notable genera of oleaginous bacteria. *Rhodococcus* sp. has received the most attention among these due to its potential to break down lignin and ultimately assimilate lignin-monomeric components into the lipid accumulation pathway [140], as well as its capacity to thrive on a variety of substrates. The *Rhodococcus opacus* DSM 1069 and pine organosolv pretreatment effluent was investigated by Wells et al. [82] as the only carbon and energy source. The results showed a maximum of 26.99 ± 2.88% of its cellular dry weight in oils to be made up of oleic, palmitic, and stearic fatty acids after 120 h at 1.5 *w*/*v*% concentration of solids. Bacteria have not been exploited for lipid synthesis as frequently as yeasts, molds, or microalgae up until now [141].

The most promising microorganisms for the synthesis of oils that are comparable to vegetable oils are oleaginous yeasts [142]. Sunflower oil, rapeseed oil, and palm oil are only a few of the common vegetable oils whose lipid profiles are comparable to those of SCO [6]. Genera like *Rhodosporidium*, *Yarrowia*, *Cryptococcus*, *Rhodotorula*, *Lipomyces*, and *Trichosporon* contain species of oleaginous yeasts, some of which may accumulate lipids up to 80% *w*/*w* of their dry cell weight [140]. For example, *Lipomyces starkeyi*, an oleaginous yeast, can grow in simple media (for example, without vitamin supplementation), perform extracellular polysaccharide degradation, and shows good tolerance to inhibitory substances like aldehydes, alcohols, and organic acids [6]. It can also afford high lipid yields from both hexoses and pentoses and re-utilize small amounts of its intracellular lipids [7].

Corn and wheat bran may be used to create SCO utilizing the oleaginous yeast *Lipomyces starkeyi ATCC 56304*, as shown by Probst and Vadlani [142]. The maize and wheat bran hydrolysates yielded the highest oil outputs, measuring 126.7 and 124.3 mg oil/g sugar, respectively. The oleaginous yeast *Lipomyces starkeyi* was examined by Di Fidio et al. [142] for its novel two-step process for converting cellulosic paper mill waste into SCO. Maximum oil productivity, single cell lipid content, lipid yield, and output were all 20.2 weight percent, 37 weight percent, 3.7 g/L, and 2.0 g/L/d, respectively.

Using *Lipomyces starkeyi* DSM 70,296 from the giant reed (*Arundo donax* L.) as a source of carbohydrates, Di Fidio et al. [6] explored an integrated hydrolysis cascade process for the production of SCO, attaining the lipid content of 30 wt% and yield values in the range of 15–24 wt%. For the purpose of SCO formation by the hydrolysate of *Arundo donax*, Zuccaro et al. [141] investigated the oleaginous yeast *Lipomyces starkeyi* and the green microalga *Chloroidium saccharophilum*, both separately and in mixed cultures. In every instance, blended cultures outperformed individual ones in terms of performance. In their overview of recent developments in oleochemical synthesis in yeast-based biorefineries, Zhang et al. [143] also discussed the use of alternate renewable feedstocks such xylose and L-arabinose.

Oleaginous yeast research has advanced significantly. The majority of bulk oleochemicals now available, however, do not have the titers, rates, or yields necessary for commercial manufacturing [143]. As an example of a successful case, it is possible to cite the conditions observed for the modified *Y. lipolytic* strain which is a good candidate for commercial production since it can manufacture FAMEs at high titers, yields, and rates of 98.9 g/L, 1.3 g/L /h, and 0.27 g/g glucose, respectively (*Saccharomyces cerevisiae*, a non-oleaginous yeast, is employed in several industrial applications because it is simple to grow and has well-established genetic tools. As a result, *S. cerevisiae* has also been used for lipid synthesis and subjected to metabolic engineering techniques [140].

Oleaginous fungi are endowed microbes because of their high oil production and have several advantages over other oleaginous microorganisms, including a high content of dry biomass, the ability to produce some special fatty acids like γ-linolenic acid in large quantities, and the ability to grow on cheap feedstocks [144]. The fatty acid profiles of 28 taxa in the *Mortierellales*, *Mucorales*, and *Umbelopsidales* were investigated by Zhao et al. [145]. Results revealed that 46% of taxa, 50% of species, and 34% of strains were oleaginous, with *Backusella* being singled out for collecting up to 59.08 2.24% of oils [145].

Because they can store large quantities of lipids (60 percent of their total lipid content produces more net oils than 50%), microalgae are known for having a high photosynthetic efficiency and higher oil production productivity [146]. *Chlorella vulgaris*, *Chlorella protothecoides*, and *Chlorella sorokiniana* are only a few of the Chlorella species strains that may collect more than 25% oil on glucose. There are, however, few investigations into the growth of microalgae on alternative carbon substrates, such as xylose, one of the principal constituents of hemicellulose [147]. Combining the oleaginous fungus *Mortierella elongata* with the marine alga *Nannochloropsis oceanica* allowed Du et al. [148] to study oil production. They produced significant amounts of triacylglycerol and total fatty acids for *N. oceanica* and *M. elongate*, with yields of around 15 and 22% of total dry weight, respectively.

Microbial oil has potential as a substitute to be integrated in food and fuel manufacture. Production has been constrained, nevertheless, because of the high cost of feedstock and the competition from cheaper oilseed crops. According to an economic analysis of the production of yeast SCO, the costs of raw materials, including feedstocks, can make up around 40% of the overall expenses [142]. Utilizing food waste streams might lead to manufacturing that is cost-competitive for the development of inexpensive SCOs [149]. These include rice straw, wheat straw, maize cobs, corn residues, corn fiber, sugarcane bagasse, sorghum bagasse, and many more biomass residues that are plentiful and appealing. Many agro-industrial wastes were evaluated by Diwan et al. [3] to SCO in order to launch a viable biorefinery.

A solution to the major environmental hazards caused by an imbalance in the generation to safe disposal ratio of agricultural and industrial processing wastewater can be found by utilizing it as a cultivation feedstock [3]. Chuppa Tostain et al. [150] investigated the growth of Aspergillus niger from residual liquid waste produced during the fermentation and distillation of sugarcane molasses (vinasse). The findings showed how well the fungus could grow on vinasse and degrade this challenging media. Additionally, the biomass of fungal organisms may be used to produce biodiesel by using the internal lipids. Vinasse has been shown to have promise for yeast and fungus growth, lipid production, and appropriateness for biodiesel needs by Hoarau et al. [151]. By co-culturing oleaginous yeast and microalgae on inexpensive substrates such as crude glycerol, wastewater, lignocellulosic biomass hydrolysate, and hydrophobic wastes, Qin et al. [152] examined methods for improving lipid synthesis.

The use of a biorefinery strategy has been recommended, where numerous co-products are generated with SCO, to assure economic viability and maximize environmental performance. Given that they serve as a platform for a variety of various intra- and extracellular products in addition to lipids, oleaginous algae and yeast processes have a significant amount of potential to profit from this approach [138]. The co-production of protein, energy co-products, glycerol and organic acids, carotenoids and other high value compounds are a few examples of the possible co-products generated with SCO.

After the lipids are treated, the microbe cake may be used as animal feed or subjected to further processing to provide amino acids or peptides, which have a variety of uses (such as biomaterials, bioplastic, and biofoam) [11].

Oleaginous yeasts have been shown to produce succinic, malic, oxalic, and citric varieties of organic acids extracellularly [138]. SCO and gluconic acid co-production was examined by Qian et al. [153] utilizing oleaginous *Cryptococcus podzolicus* DSM 27192. The findings indicated a rise in the economic worth of the production of biodiesel from microbial lipids. The outcomes of the suggested co-production method may also serve as a model for other co-production systems. Gao et al. [154] investigated the synthesis of succinic acid from crude glycerol by an engineered yeast, *Y. lipolytica*. The findings showed that *Y. lipolytica* is a potentially useful microbial factorial cell for a very effective method of resolving environmental issues associated with the manufacturing of value-added goods. Yeast strains including *Rhodotorula glutinis, Rhodotorula rubra, Sporobolomyces roseus, Phaffia rhodozyma*/*Xanthophyllomyces dendrorhous*, and *Sporobolomyces ruberrimus* can collect valuable carotenoids like β-carotene, torulene, and astaxanthin [152,155].

In order to produce both commodity and specialty goods, such as whey protein concentrate, antioxidants, ethanol, tartrate salts, and microbial oil, Kopsahelis et al. [156] investigated the integrated refining of cheese whey and wine lees. Using typical sunflower-based biodiesel production plants, Leiva-Candia et al. [157] investigated the synthesis of value-added by-products (such as protein isolate, antioxidant-rich extracts, and microbial oil). By adopting a biorefinery strategy and valuing coproduct streams, the environmental and financial viability of SCO can be increased. The protein percentage appears to be crucial in establishing the minimum oil selling price and environmental effect among the different coproduct options [138].

## 5. Microbial Lipid-Based Products

We can consider the application of microbial lipids produced by bacteria, yeasts, filamentous fungi, and microalgae in four main areas: the biofuel, pharmaceutical, cosmetics, and food and feed sectors. The quest to establish fourth-generation biofuels has fostered basic and applied research as an alternative to renewable energy sources, mainly when associated with agricultural waste [158,159]. Special attention has yet to be given to lignocellulosic sources and crude glycerol, a by-product of biodiesel production, which may generate an associated production chain and become technologically and economically viable shortly [160]. In addition to its use as a biofuel, there is the prospect of using it as an automotive lubricant additive [137] and wax ester [161].

The application of lipids and all their molecules associated with the pharmaceutical industry is a topic of great interest and much research. Using a stabilizing agent for pharmaceutical emulsions is a promising application [162]. Similarly, microbial lipids and their specificities allow a wide field of applications. Some microbial lipids are sophorolipids (SLs), which due to their hydrophobic characteristic, can play an essential role in formulations, helping drug release. Otherwise, a spermicidal activity of microbial SLs was observed in humans [163]. Other pharmacological activities have also been attributed to PLs, such as immune-response modulators and anti-inflammatory and anti-viral agents [164]. The performance as an anti-cancer and antimicrobial agent was also attributed to microbial glycolipids [165].

The role of microbial lipids in the cosmetics industry can be established in two different ways: the functional properties, acting as an emulsifier, dispersant, or thickener [166] and the physiological properties, with the action of skin cleaners, photoprotector [167], skin conditioner [168], antioxidant and anti-aging effects. Complementarily, positive effects in the treatment of cellulite, in the synthesis of collagen, and the treatment of wrinkles were also observed. Thus, due to their structural similarity with skin lipids, permeability is an essential feature of microbial glycolipids, enabling an efficient functional or physiological action [166,168].

Although several studies were directed to producing polyunsaturated fatty acids from microalgae [169], filamentous fungi and yeasts have a prominent role in the production of microbial lipids for food applications, which can mainly be directed to the production of dietary supplements such as essential fatty acids [109] or oils rich in arachidonic acid [170]. One of the first applications of microbial lipids was in the early 1990s as a substitute for cocoa butter in the manufacture of chocolate [171]. In 2001, there was an important advance in the consolidation of the inclusion of microbial lipids as a food supplement. The Food and Drug Administration (FDA) has recognized “Oil of Javanicus” as safe (GRAS) for application in infant formulas [84]. Thus, it is estimated that by 2010 more than 24 million babies consumed food containing microbial lipids [171]. Complementarily, another critical step in using microbial lipids is the association with nanotechnology to improve stability during storage, facilitate incorporation into food matrices or increase bioavailability after ingestion [172].

Several companies such as DSM (Switzerland), Cargill Alking Bioengineering (China), Nestle S.A, Fermentalg (France), Algomed (Germany), Phytolutions (Germany), Parry nutraceuticals (India), Oilgae (India), Algatechnologies (Israel), Sunchlorella (Japan), Algae Technology Solutions (Mexico), Fitoplancton Marino (Spain), Simris (Sweden), Vedan (Taiwan), Aurora Algae (United States), Algaeon (United States), Altech Algae (United States), and Algae to Omega Holdings (United States) produce or use microbial lipids [171]. Currently, DSM is the company with the most significant number of commercial microbial products [171]. In Table 4 it is possible to observe some commercial products with a lipid base of microorganisms.

## 6. Perspectives for Microbial Lipid-Based Products

Perspectives for microbial lipids must be established mainly by technical and economic feasibility. The reduction of production cost is a preponderant factor. The effect of the cost of production is linked to the purpose of the product. For example, products with higher added values tend to be less influenced in the cosmetics and pharmaceuticals industry [11]. However, several studies indicate that using agricultural residues as a growth medium for oleaginous microorganisms can be a viable and sustainable technology, especially for abundant raw materials, such as lignocellulosic residues, except for the technical obstacles that still need to be addressed, such as the generation of inhibitory products during treatment [39]. As seen, the metabolism of these microorganisms is highly adaptable, enabling different products of interest [32]. Thus, the number of companies that operate in a consolidated manner in producing microbial lipids indicates this market’s potential.

## 7. Conclusions

In this review, it was possible to observe that many microorganisms can accumulate lipids as intracellular storage compounds, such as yeasts, fungi, molds, and microalgae. Briefly, lipids formed by microorganisms can be formed and accumulated intracellularly or in chloroplasts. Such oils show a fatty acid profile similar to that of vegetable oils (mostly used for the production of first-generation biodiesel). In addition, the fatty acid profile of SCO features essential fatty acids, so-called polyunsaturated fatty acids (PUFAs), not synthesized by mammals and obtained from food. This creates a scenario of greater accessibility for the population to these nutrients, which are significant nutrients for human health. In addition, one of the main obstacles for SCO biorefineries to produce good results is the lack of more detailed technical studies, as these are still limited, with the need for technical-economic evaluation of the use of oleaginous microorganisms for the production of SCO. The value of USD3000/ton was verified for microbial oil, similar to the price of cocoa butter, which demonstrates the high potential of this raw material. Thus, this technology, in addition to making it possible to increase the volume of quality lipid raw material which is available and of great commercial interest, creates space for a new consumer market, in addition to new products based on the new oils produced.

## Figures and Tables

**Table 1:** Microorganisms with potential application for the production of lipids.

Microorganisms with High Potential	Microorganisms with Low Potential
*S. occidentalis* [13]	*P. Silvicola* [13]
*M. pulcherrima* [13]	*C. intermedia* [13]
*L. elongisporus* [13]	*P. pertersonii* [13]
*W. lipofer* [13]	*C. lusitaniae* [13]
*Y. lipolytica* [14]	*K. phaffii* [13]
*H. californica* [13]	*S. roseus* [13]
*P. anomala* [13]	*P. augusta* [13]
*T. delbrueckii* [13]	*C. bombicola* [13]
*H. beyerinckii* [13]	*K. apiculate* [13]
*C. tropicalis* [13]	*C. glabatra* [13]
*R. toruloides* [15]	*T. elliptica* [16]
*L. starkeyi* [17]	*B. braunii* [18]
*C. curvatus* [19]	*R. glutinis* [20]
*F. oxysporum* [21]	*Microsphaeropsis* sp. [22]
*R. opacus PD630* [23]	*R. opacus DSM 1069* [24]

**Table 2:** SCO production by different microorganisms.

Source of SCO Production	Microorganism Used	Substrate Used	Yield of SCO Production (g/L)	Advantages	Disadvantages	References
Algae	*Chlorella vulgaris*, *Chlorella sorokiniana*, *Botryococcus braunii*, *A. protothecoides SAG 211-7a*, *Chlorella* sp.	Glucose or CO_2_	10–100	Fast growth rate, high lipid-content	High production cost, contamination issues	[90,91,92,93,94]
Yeast	*Yarrowia lipolytica*, *Cryptococcus* sp., *Cryptococcus curvatus*,	Glucose or agro-industrial waste	30–60	Can utilize a variety of substrates, easy to manipulate	Low lipid content	[95,96,97,98,99]
Fungi	*Mortierella alpina*, *Mucor circinelloides*, *Mortierella alpina*, *Aspergillus*, *Penicillium*, *Fusarium and Alternaria*, *Cunninghamella echinulata*	Glucose or plant oil	60–80	High lipid content, can produce polyunsaturated fatty acids	Slow growth rate	[100,101,102,103]
Bacteria	*Rhodococcus opacus*, *Sterculia foetida, E. coli and Acinetobacter baylyi*	Plant oil or glucose	40–80	High lipid content, can utilize a variety of substrates	Slow growth rate, low lipid productivity	[104,105,106,107,108]

**Table 3:** Main strategies used for the development of strains.

Strategies	Realization Mechanism	Example	Bibliography
Mutation	By creating a mutant strain with the use of physical and chemical mutagens, strain improvement is achieved.	An important commercial version of tetracycline is 6-methyl tetracycline, which is produced by a mutant strain of Streptomyces aureofaciens.	[120]
Recombination	The process that combines two genotypes to create a new genotype is known as genetic recombination. Effective strain improvement requires careful mutagenesis to maintain genetic diversity as well as the employment of sister strains, divergent strains, and ancestor crosses at predetermined intervals.	Recombination after meiotic Return-To-Growth in a sterile polyploid hybrid yeast.	[121]
Protoplast fusion	Protoplast transformation or protoplast fusion is one of the most significant developments in recent years. Protoplast has a very strong negative charge on its surface, making fusion difficult.	Sclerotium rolfsii, a phytopathogenic fungus, protoplast isolation and fusion (sacc.)	[122]
Gene technology	In vitro recombination and gene manipulation are both part of gene technology. These techniques allow for the replication of specified DNA sequences inside prokaryote or eukaryote organisms.	Genetically modified filamentous fungus, bacteria, and yeast are being used in the food business to produce terpenes.	[123]

**Table 4:** Microbiological lipid-based commercial products.

Product	Source	Company/Country	Applications	Reference
Javanicus oil	*Mucor javanicus*	J & E Sturge, North Yorkshire, UK	Oil rich in linolenic fatty acid, used as a substitute for evening primrose oil	[173]
DHASCO-B	*microalgae Crypthecodinium cohnii*	DSM, Grenzach, Switzerland	Benefits for the optimal development of the child’s brain, eyes and nervous system. Support a healthy pregnancy	[174]
SUNTGA40S in Japan and Cabio oil in China	Produced by *Crypthecodinium cohnii and various strains and species of thrautochyrids*	Produced by Suntory, Osaka, Japan and sold in China by Cargill	Enrichment of infant formulas	[175]
life’s OMEGA	*Crypthecodinium cohnii and Schizochytrium* sp.	DSM, Grenzach Switzerland	Prevention of cardiovascular diseases	[171]
Eicooil	*microalgae Y. lipolytica genetically modified*	Qualitas Health Ltd., Jerusalem, Israel	Benefits for blood pressure, platelet aggregation and various inflammatory process	[176]
Simris Algae ômega-3	*Chlorella vulgaris*	Simris, Simrishamn, Sweden	Prevention of cardiovascular diseases	[177]
BioAstin SCE5 and BioAstin SCE10	*Haematococcus pluvialis*	Parry Nutraceuticals, Chennai, India	antioxidant effects	[178]

## Data Availability

Not applicable.
